# Contrast-enhanced ultrasound features of primary renal Ewing-like undifferentiated small round cell neoplasm: a case report

**DOI:** 10.3389/fonc.2026.1902656

**Published:** 2026-09-03

**Authors:** Xueling Tang, Jie Xue

**Affiliations:** 1 School of Medical Imaging, Shandong Second Medical University, Weifang, China; 2 Department of Ultrasound Medicine, Yuhuangding Hospital, Yantai, China

**Keywords:** contrast-enhanced ultrasound, Ewing-like sarcoma, needle biopsy, renal tumor, ultrasound

## Abstract

This article reports on the diagnosis and treatment of a patient with a renal Ewing-like undifferentiated small round cell neoplasm. The patient, a 56-year-old man, presented to our hospital on March 14, 2025, complaining of “blood clots in the urine for 2 days” and “a mass in the right kidney detected on physical examination 2 days prior.” On March 21, 2025, he underwent a “right renal mass needle biopsy” under local infiltration anesthesia. Postoperative pathology: (right renal mass) malignant tumor with necrosis; immunohistochemical findings consistent with renal Ewing-like undifferentiated small round cell neoplasm. Postoperative follow-up: The patient’s condition progressed after six cycles of chemotherapy. Immunotherapy combined with anti-angiogenic targeted therapy was planned but proved ineffective; the patient ultimately died of multiple organ failure.

## Introduction

**Figure 1:**
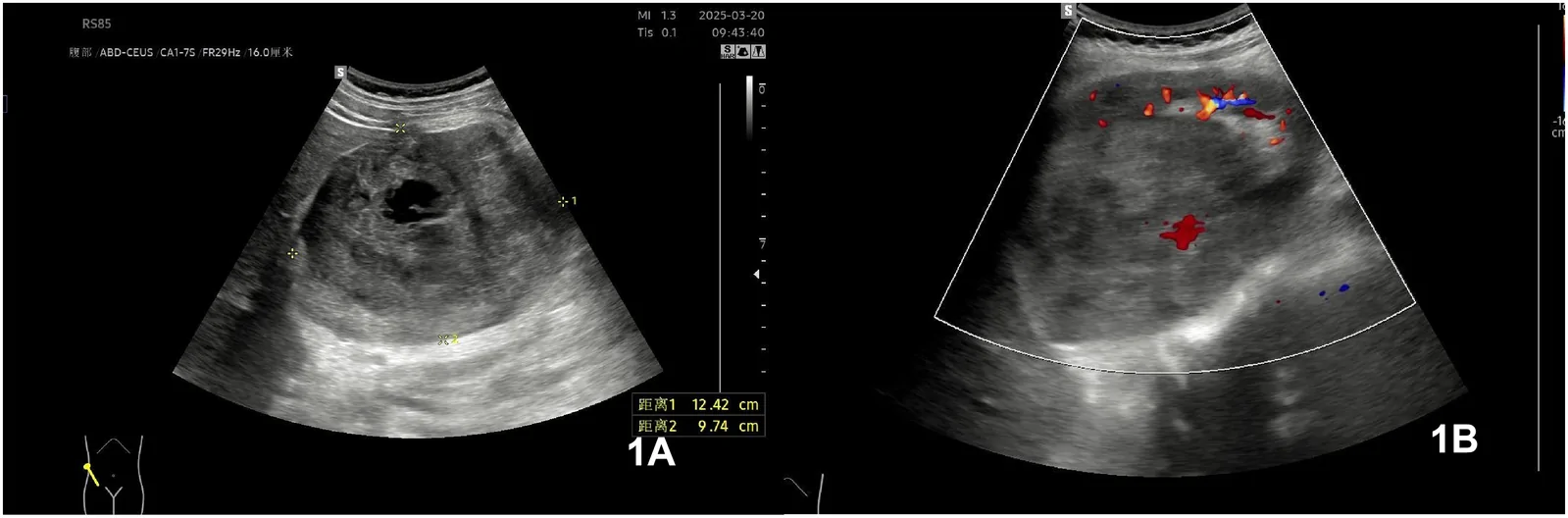
**(A, B)** Male, 56 years old, Ewing-like undifferentiated small round cell neoplasm of the kidney. Ultrasound examination **(A)** shows a hypoechoic mass in the right renal parenchyma, approximately 12.6 * 10.0 cm in size, with an unclear boundary, an irregular shape, and uneven internal echo. Color Doppler flow imaging (CDFI): A small amount of blood flow signal can be seen within the mass **(B)**.

**Figure 2:**
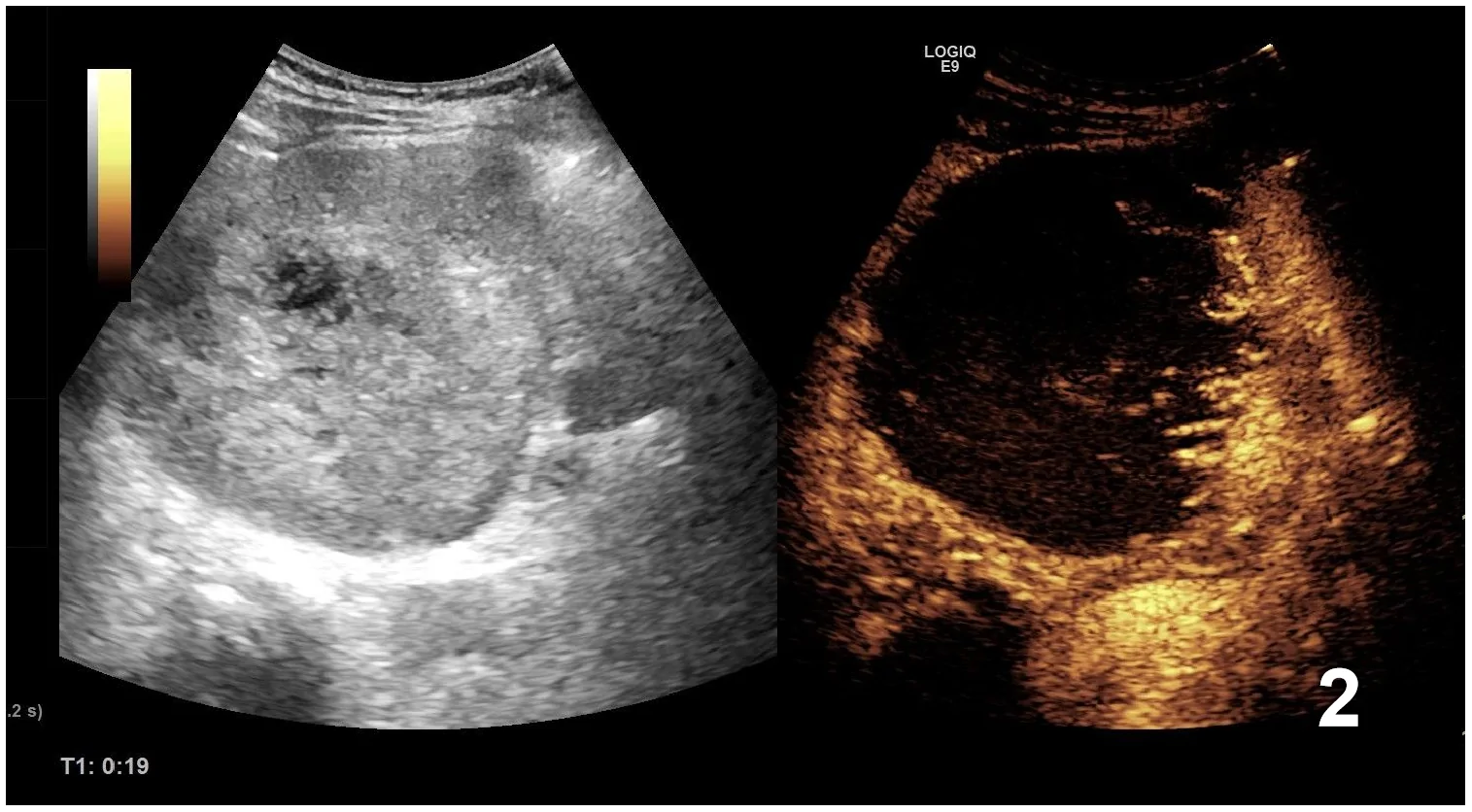
Male, 56 years old, Ewing-like undifferentiated small round cell neoplasm of the kidney. CEUS image of the kidney: The lesion in the right kidney shows delayed enhancement, with the enhancement intensity being lower than that of the surrounding renal parenchyma. There are scattered large areas of perfusion defects within the lesion.

The patient, a 56-year-old man, was admitted for “blood clots in the urine for 2 days” and “a mass detected in the right kidney during a physical examination 2 days ago.” Ultrasound revealed a hypoechoic mass in the right renal parenchyma, measuring approximately 12.6 *10.0 cm, with indistinct borders, an irregular shape, and heterogeneous internal echogenicity. Color Doppler flow imaging (CDFI) showed minimal blood flow signals within the mass (**Figures 1A, B**). Contrast-enhanced ultrasound (CEUS) was performed using a GE E11 ultrasound scanner with a C1–6 convex array transducer (1.0–6.0 MHz). A 2.5 ml bolus of SonoVue suspension was administered intravenously, followed by a 5 ml bolus of saline to flush the line. Imaging was performed in low mechanical index mode (MI = 0.13), and dynamic imaging was recorded throughout the entire process. The perfusion characteristics of contrast agent uptake and washout were observed sequentially during the cortical, parenchymal, and delayed phases. The mass began to enhance at 19 seconds, reached its peak at 31 seconds, and exhibited delayed enhancement. The degree of enhancement was less than that of the surrounding normal renal parenchyma. Scattered large areas of perfusion defects were visible within the lesion, suggesting a possible malignant lesion (**Figure 2**).

**Figure 3:**
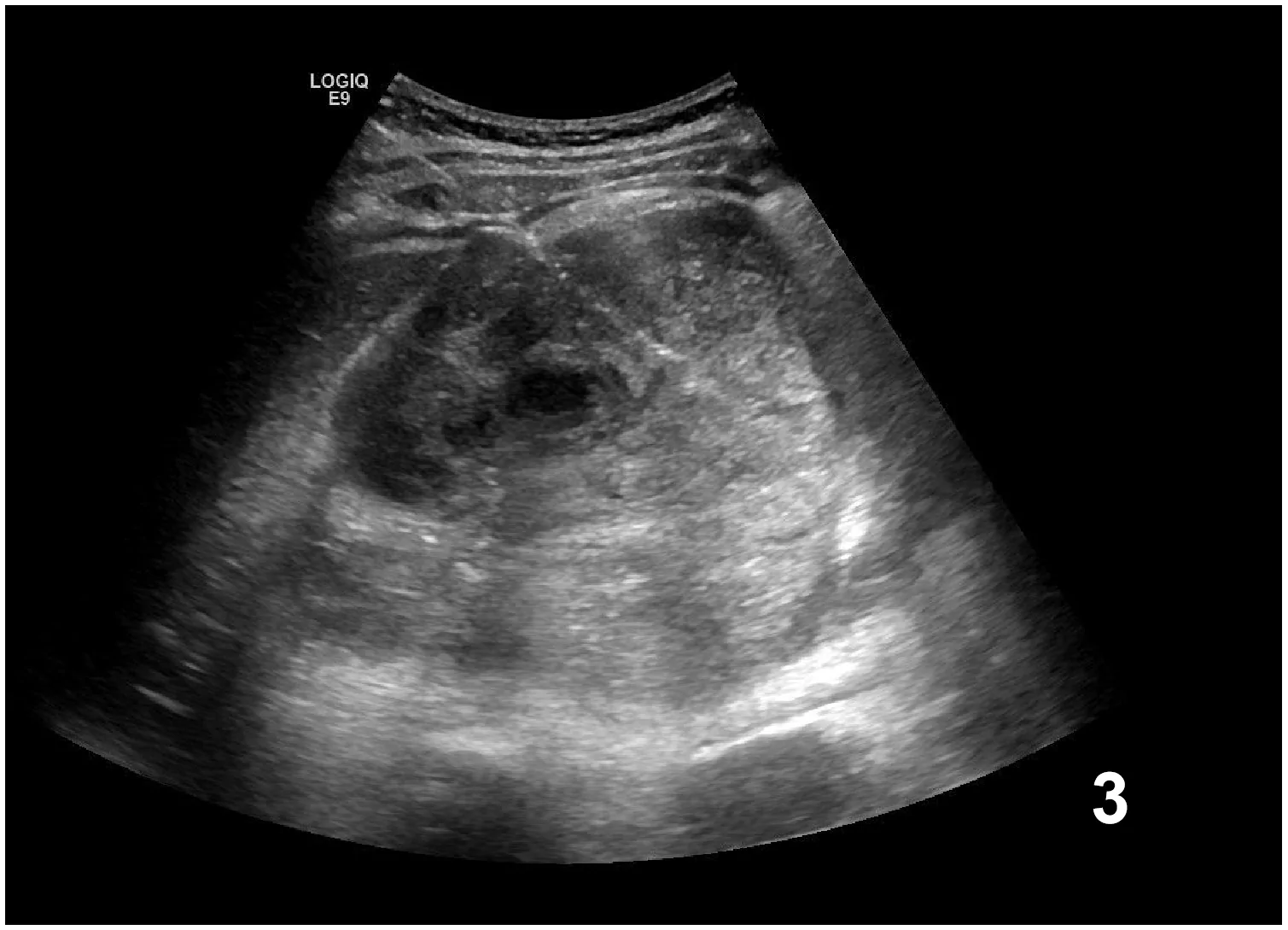
Male, 56 years old. Ewing-like undifferentiated small round cell neoplasm of the kidney. Ultrasound-guided biopsy.

**Figure 4:**
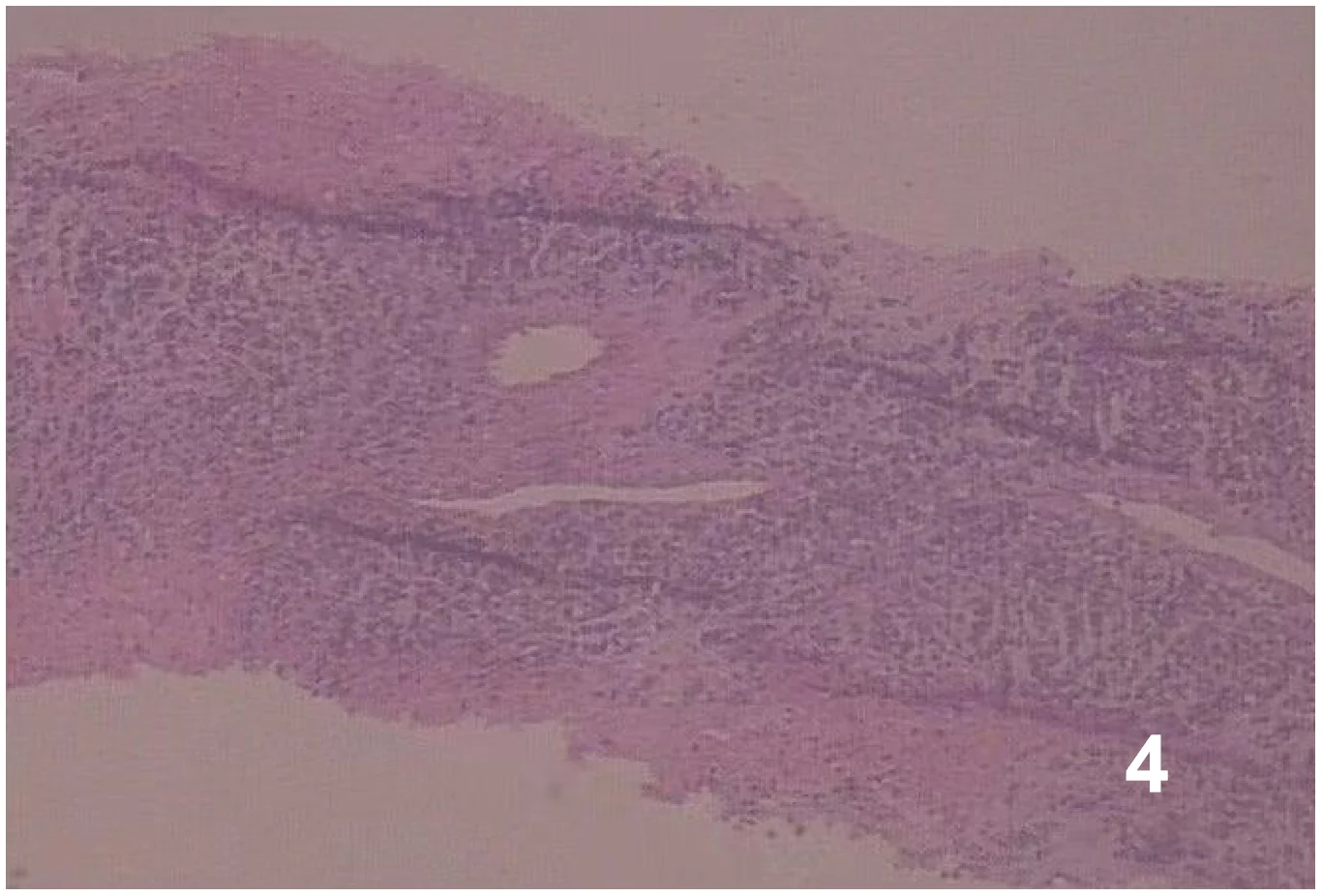
Male, 56 years old, Ewing-like undifferentiated small round cell neoplasm of the kidney. Pathological image: Under the microscope, small round tumor cells of consistent size can be seen, with round or oval cell nuclei, fine chromatin, scarce cytoplasm, cells arranged in nests, sheets or diffusely, with unclear boundaries, and pseudo-daisy-like structures can be observed (HE, ×100).

An “ultrasound-guided needle biopsy of a right renal mass” was performed under local infiltration anesthesia (**Figure 3**), Postoperative pathology (**Figure 4**): (Right renal mass) Malignant tumor with necrosis; microscopically, small, round tumor cells of relatively uniform size are visible, with round or oval nuclei, fine chromatin, and scant cytoplasm. The cells are arranged in nests, sheets, or diffusely, with indistinct borders; pseudo-rosette structures are visible. Immunohistochemistry: CD99(+), NKX2.2(-), CgA(-), CK(-), INI-1(+), MelanA(-), EMA(-), S-100(-), desmin(-), myogenin(-). Consistent with a Ewing-like undifferentiated small round cell neoplasm of the kidney. Considering the patient’s creatinine levels, the patient received alternating-scheme chemotherapy, undergoing treatment with vincristine, doxorubicin, and cyclophosphamide from April 21, 2025, to May 6, 2025. On May 14, 2025, the patient received etoposide plus ifosfamide. After six cycles, the patient’s condition progressed, and on September 5, 2025, treatment was switched to a combination of immunotherapy and anti-angiogenic targeted therapy. The treatment was ineffective, and the patient died of multiple organ failure on April 2, 2026.

## Discussion

According to the “WHO Classification of Bone and Soft Tissue Tumors (5th Edition, 2020)” (1), cases that are morphologically consistent with the Ewing spectrum and lack an EWSR1-ETS fusion are collectively referred to as undifferentiated small round cell sarcomas of the Ewing spectrum. These tumors originate from primitive mesenchymal stem cells and are classified into four molecular subtypes: classic Ewing sarcoma, CIC-rearranged sarcoma, BCOR-aberrant sarcoma, and EWSR1-non-ETS fusion small round cell sarcoma (2). These tumors predominantly affect adolescents and young adults. Clinically, they primarily present as painless localized masses that progressively enlarge; they are prone to early distant metastasis, are highly aggressive, and have a relatively high likelihood of local recurrence (3). The morphology of this case closely resembles that of Ewing-spectrum lesions; however, due to limited specimen quality, molecular testing could not be performed in full, and evidence of characteristic fusion genes was lacking. For such cases, the term “Ewing-like undifferentiated small round cell neoplasm” is uniformly adopted as the morphological descriptive diagnosis; this nomenclature has been applied in reports of similar lesions from multiple centers.

Ultrasound plays an important role in the diagnosis of malignant kidney tumors, primarily presenting as hypoechoic, lobulated lesions with indistinct borders and posterior echo enhancement; CDFI reveals abundant blood flow, with multiple short, strip-like blood flow patterns penetrating the tumor (4). Since Ewing-like undifferentiated small round cell tumor is often accompanied by marked cystic changes, necrosis, or hemorrhage (5), conventional ultrasound may be unable to distinguish between cystic and necrotic areas. However, CEUS can clearly display the contrast-enhanced borders and internal perfusion differences of the lesion in real time, effectively distinguishing solid viable tissue from non-perfused necrotic areas. This allows for the planning of needle insertion paths based on the active lesion regions highlighted by contrast enhancement, enabling targeted tissue sampling and improving the accuracy of pathological diagnosis. This approach is of great value for assessing the extent of tumor invasion and its activity (6), and provides important imaging evidence for distinguishing renal Ewing-like undifferentiated small round cell tumor from other benign and malignant tumors.

According to the literature (7, 8), renal Ewing sarcoma/primitive neuroectodermal tumors primarily manifest on CEUS as annular enhancement and heterogeneous enhancement, with synchronous enhancement being the predominant pattern. Other common malignant renal tumors, such as clear cell renal cell carcinomas (ccRCCs), are characterized by an abundance of coarse, irregular, and tortuous blood vessels accompanied by arteriovenous fistulas, as well as rapid tumor growth and a tendency toward ischemic necrosis; consequently, they typically exhibit early flow, high enhancement, and heterogeneous enhancement. The patient in this case presented with low enhancement and delayed enhancement, suggesting that the degree and timing of enhancement may be relevant to the diagnosis of Ewing-like undifferentiated small round cell tumor. In addition, it is necessary to differentiate this condition from other rare kidney tumors, such as renal lymphoma,sarcomatoid renal carcinoma and metastatic small round cell malignancy.

Treatment for Ewing-like undifferentiated small round cell tumor is currently primarily modeled after that for Ewing sarcoma and mainly involves various combinations of surgery, radiation therapy, and chemotherapy; however, there are currently no unified treatment guidelines either domestically or internationally (9). Studies have shown (10) that nearly all chemotherapy regimens are based on combinations of the most potent drugs, such as doxorubicin, cyclophosphamide, vincristine, etoposide, ifosfamide, and dactinomycin; moreover, undifferentiated small round cell neoplasms are highly resistant to chemotherapy and have a poor prognosis. Therefore, there is a need to further improve the diagnostic accuracy of this disease and refine molecular genetic testing in order to develop individualized, precision treatment regimens.

In summary, primary renal Ewing-like undifferentiated small round cell neoplasms are clinically rare. Preoperative imaging can help distinguish between benign and malignant tumors, but a definitive diagnosis still requires pathological and immunohistochemical findings. The characteristics of CEUS low enhancement and delayed enhancement may aid in diagnosis and are of significant value in distinguishing between viable and necrotic tumors, as well as in guiding biopsies. CEUS holds unique potential for use in special patient populations, such as children, patients with impaired renal function, and those with allergies to CT or MRI contrast agents.

## Data Availability

The original contributions presented in the study are included in the article/Supplementary Material. Further inquiries can be directed to the corresponding author.
